# ﻿Two new species of *Hemiptarsenus* Westwood (Hymenoptera, Eulophidae) from China, with a key to Chinese species

**DOI:** 10.3897/zookeys.1103.85228

**Published:** 2022-05-26

**Authors:** Jun-Jie Fan, Cheng-De Li

**Affiliations:** 1 School of Forestry, Northeast Forestry University, Harbin, 150040, China Northeast Forestry University Harbin China

**Keywords:** Chalcidoidea, Eulophinae, parasitoid, taxonomy

## Abstract

Two new species of *Hemiptarsenus* Westwood, *H.tianshuiensis***sp. nov.** and *H.longjiangensis***sp. nov.**, are described from China. New distributional data for *H.jilinus* Tao, 2021 are provided, and a key to Chinese species of the genus is given based on females.

## ﻿Introduction

The genus *Hemiptarsenus* (Hymenoptera, Eulophidae) was erected by Westwood (1833) with *Hemiptarsenusfulvicollis* Westwood as the type species. This genus is mainly distributed in the Palaearctic region, where 17 species were recorded. Currently the genus contains 34 valid species worldwide: 33 species were recorded in the Universal Chalcidoidea Database (Noyes 2019), and one species was described recently by Tao et al. (2021). Eight species are known from China: *H.fulvicollis* Westwood, 1833, *H.jilinus* Tao, 2021, *H.ornatus* (Nees, 1834), *H.strigiscuta* Zhu, LaSalle & Huang, 2000, *H.tabulaeformisi* Yang, 2015, *H.unguicellus* (Zetterstedt, 1813), *H.varicornis* (Girault, 1913), and *H.zilahisebessi* Erdös, 1951 (Sheng et al. 1989; Lee 1990; Zhu et al. 2000; Xu et al. 2001; Zhu and Huang 2002; Yang et al. 2015; Tao et al. 2021). Members of this genus are mainly larval or nymphal parasitoids of Diptera (Agromyzidae, Ephydridae), Hemiptera (Coccidae), Lepidoptera (Cosmopterigidae, Elachistidae, Gracillariidae, Lyonetiidae, Momphidae, Nepticulidae, Pyralidae, Yponomeutidae), Coleoptera (Curculionidae), and Hymenoptera (Tenthredinidae) (Yang et al. 2015).

*Hemiptarsenus* species can be recognized by the following combination of characteristics: funicle 4-segmented in females and with three branches in males; apex of scape extending above level of vertex; notauli incomplete; mesoscutellum without sublateral grooves; fore wing costal cell narrow, at least 10 times as long as wide.

This study describes two new species of the genus. New distributional data for *H.jilinus* Tao, 2021 and a key to females of all species of the genus are provided.

## ﻿Material and methods

All specimens were collected by sweeping or yellow-pan trapping, and they were dissected and mounted in Canada balsam on slides following the method of Noyes (1982) or mounted on cards. Slide-mounted specimens were photographed with a digital CCD camera attached to an Olympus BX51 compound microscope. Specimens on card were photographed with an Aosvi AO-HK830-5870T microscope. Measurements were made using the built-in software of the Aosvi AO-HK830-5870T. The quality of these photos was improved by using Helicon Focus 7 and Adobe Photoshop 2020.

Terminology follows the Hymenoptera Anatomy Consortium (2022) for most body parts except the callus, which follows Gibson (1997). The following abbreviations are used:

**F1–4** flagellomeres 1–4;

**MV** marginal vein;

**OOL** minimum distance between a posterior ocellus and corresponding eye margin;

**PMV** postmarginal vein;

**POL** minimum distance between posterior ocelli;

**SMV** submarginal vein;

**STV** stigmal vein.

All type material is deposited in the insect collections at Northeast Forestry University (**NEFU**), Harbin, China.

## ﻿Results

### ﻿Key to Chinese species of *Hemiptarsenus* Westwood based on females

**Table:** 

1	Mesoscutellum longitudinally sculptured (e.g. Fig. 20)	**2**
–	Mesoscutellum reticulate (e.g. Figs 5, 15)	**4**
2	Propodeum, axillae and metascutellum smooth	***H.strigiscuta* Zhu, LaSalle & Huang**
–	Propodeum, axillae and metascutellum reticulate	**3**
3	Mesoscutellum orange-yellow or yellow; propodeum without median carina and plicae	***H.ornatus* (Nees)**
–	Mesoscutellum dark metallic green; propodeum with complete median carina and plicae	***H.jilinus* Tao**
4	Metascutellum predominantly smooth	**5**
–	Metascutellum predominantly reticulate	**8**
5	Clava dark brown basally and pale yellow or white apically	***H.varicornis* (Girault)**
–	Clava completely dark brown	**6**
6	Mesoscutum metallic green with transverse yellow patch; length of propodeum at most half the length of mesoscutellum, plicae and median carina absent	***H.zilahisebessi* Erdös**
–	Mesoscutum completely metallic green; length of propodeum at least 0.7× as long as mesoscutellum, plicae and median carina present	**7**
7	Axillae mostly smooth; midlobe of mesoscutum with 3 pairs of setae (Fig. 5)	***H.tianshuiensis* sp. nov.**
–	Axillae reticulate; midlobe of mesoscutum with 2 pairs of setae	***H.unguicellus* (Zetterstedt)**
8	PMV shorter than or at most as long as STV; disc of fore wing slightly clouded	***H.fulvicollis* Westwood**
–	PMV at least 1.9× as long as STV; fore wing hyaline	**9**
9	Gaster with a large median longitudinal black patch from base to apex, margins of tergites 1–5 yellow; plicae complete (Fig. 10)	***H.longjiangensis* sp. nov.**
–	Gaster predominantly dark brown; plicae short and incomplete, only present in posterior 1/5	***H.tabulaeformisi* Yang**

#### ﻿ Hemiptarsenus tianshuiensis sp. nov.

Taxon classificationAnimaliaHymenopteraEulophidae

8952CB97-4370-55EA-ABB4-933667C93062

http://zoobank.org/E29DB39C-1E3B-4213-B1F4-61A82EAF6891

Figs 1–3 , 4–8

##### Type material.

***Holotype***, ♀ [NEFU; on card], China, Gansu Province, Tianshui City, Maiji District, Maijishan National Geopark, 23.VII.2020, Jun-Jie Fan, by sweeping. ***Paratypes***: 2♀1♂; [1 ♀ on slide, 1 ♀1♂on cards], same data as holotype.

**Figures 1–3.**
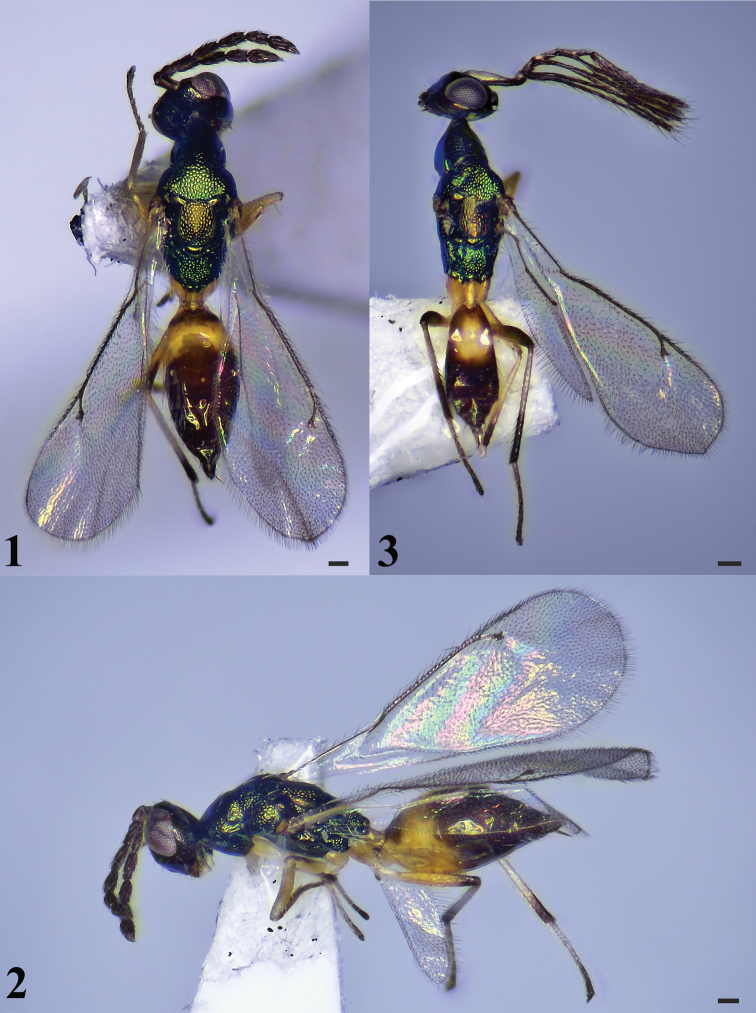
*Hemiptarsenustianshuiensis* sp. nov., female, holotype (**1, 2**), male, paratype (**3**) **1** habitus in dorsal view **2** habitus in lateral view **3** habitus in dorsal view. Scale bars: 100 μm.

##### Diagnosis.

Antennae dark brown with ventral surface of scape yellow. Metascutellum mostly smooth with anterior area reticulate. Propodeum about 0.7× as long as mesoscutellum measured medially, and strongly reticulate, median carina and plicae present. Mid and hind leg tibiae yellowish-white with apical 1/3 dark brown, metafemur yellow with apical 1/4 dark brown. Petiole yellow. Gaster dark brown with a transverse yellow patch near base.

##### Description.

**Female.** Length 1.8–2.0 mm (2.0 mm) mm, fore wing length 1.6–1.8 mm (1.8 mm) mm. Head dark metallic green. Eyes red-brown. Ocelli pale yellow. Scape yellow except dorsal surface dark brown, pedicel and flagellum dark brown. Mesosoma dark metallic green except mesoscutellum with golden-green tinge. Petiole yellow. Gaster dark brown with a transverse yellow patch near base. Fore leg mostly yellowish white with tarsomeres 1–3 brown, tarsomere 4 dark brown; mid leg with coxae and trochanters yellowish white, femur yellow with apical 1/2 brown on dorsal surface, tibiae yellowish-white with apical 1/3 dark brown, tarsomeres 1 and 2 yellowish white and tarsomeres 3 and 4 dark brown; hind leg similar to mid leg with femur apical 1/4 dark brown.

**Figures 4–8.**
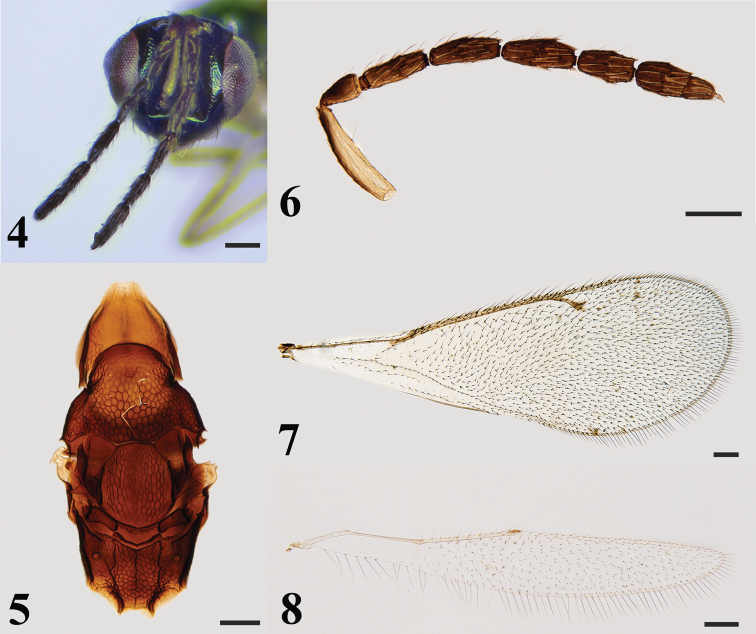
*Hemiptarsenustianshuiensis* sp. nov., female, holotype (**4**), paratype (**5–8**) **4** head in frontal view **5** mesosoma in dorsal view **6** antenna **7** fore wing **8** hind wing. Scale bars: 100 μm.

***Head*** (Fig. 4) 1.2–1.3× (1.2×) as wide as high in frontal view and 1.9–2.1× (2.0×) as wide as long in dorsal view, micro-reticulate. POL 2.0× OOL. Eyes with short and dense setae. Malar sulcus present, malar space 0.34× eye height. Mandibles quadridentate. Antennae (Fig. 6) with scape slender and cylindrical, 4.3–4.6× (4.6×) as long as wide, extending far above vertex; pedicel 1.9–2.0× (1.9×) as long as wide and scape 2.4–2.6× (2.5×) as long as pedicel; funicle 4-segmented, F1 2.9–3.2× (3.0×) as long as wide and 1.3–1.4× (1.4×) as long as pedicel, F2 2.9–3.1× (2.9×) as long as wide, F3 and F4 2.6–2.8× (2.6×) and 2.0–2.1× (2.0×) as long as wide respectively; clava 2-segmented, 2.5–2.7× (2.6×) as long as wide, the first clavomere 1.8–1.9× (1.8×) as long as the second.

***Mesosoma*** (Figs 1, 5) 2.0–2.2× (2.1×) as long as wide. Pronotum shorter than mesoscutum, reticulate. Notauli inconspicuous. Mesoscutum strongly reticulate, midlobe of mesoscutum with three pairs of long setae. Axillae mostly smooth and separated from each other. Mesoscutellum 1.2–1.3× (1.2×) as long as wide, shorter than mesoscutum, strongly reticulate, and with two pairs of long setae. Metascutellum mostly smooth with anterior area reticulate. Propodeum about 0.7 × as long as length of mesoscutellum measured medially, strongly reticulate, median carina and plicae present; spiracle separated from metanotum by a distance longer than diameter of spiracle; each propodeal callus with 13 setae. Prepectus with coarse reticulate sculpture. Metacoxa reticulate on dorsal surface.

***Wings*.** Fore wing (Fig. 7) 2.7–2.9× (2.9×) as long as wide. Costal cell 13.7–14.0× (14.0×) as long as wide, with a row of black setae on dorsal surface. SMV with five setae on dorsal surface. Cubital vein straight at base. Speculum small, closed posteriorly. MV 1.3–1.4× (1.3×) as long as PMV. PMV 2.0–2.1× (2.0×) as long as STV. Hind wing (Fig. 8) about 6.4–6.9× (6.9×) as long as wide.

***Metasoma*** (Fig. 1) 1.1–1.2× (1.1×) as long as length of mesosoma. Petiole longer than wide in dorsal view. Gaster ovate, 2.2–2.4× (2.3×) as long as wide. Ovipositor exserted beyond apex of gaster.

**Male** (Fig. 3). Similar to the female. Body length 1.6 mm, fore wing length 1.5 mm. Head 1.2× as wide as high in frontal view and about 1.9× as wide as long in dorsal view. POL 2.43× OOL. Malar space 0.5× eye height. Antennae dark brown with ventral surface of scape yellow, funicle with three long branches, with long setae. Relative measurements (length: width): scape = 33: 8; pedicel = 10: 7; F1 = 10: 6; F2 = 20: 4; F3 = 22: 4; F4 = 40: 7; clava = 40: 7. Fore wing 3.1× as long as wide. Hind wing about 7.1× as long as wide. MV 1.3× as long as PMV. PMV 2.0× as long as STV. Metasoma almost as long as mesosoma. Petiole 1.7× as long as wide in dorsal view. Gaster ovate, 1.9× as long as wide.

##### Host.

Unknown.

##### Distribution.

China (Gansu).

##### Etymology.

The specific epithet refers to the location of the type locality in Tianshui City.

#### ﻿ Hemiptarsenus longjiangensis sp. nov.

Taxon classificationAnimaliaHymenopteraEulophidae

09254467-3563-54D5-8374-48890E931C14

http://zoobank.org/F4B950D8-204A-45CA-8CA6-46A96B867426

Figs 9 , 10–15

##### Type material.

***Holotype***, ♀ [NEFU; on card], China, Heilongjiang Province, Yichun City, Dailing District, Liangshui National Nature Reserve, 30–31.VIII. 2019, Wen-Jian Li, Ting-Ting Zhao and Shu-Chen Deng, by yellow-pan trapping. ***Paratypes***: 1♀ [on slide], China, Heilongjiang Province, Shangzhi City, Maoershan Town, 9.VII.2015, Ye Chen and Chao Zhang, by sweeping; 2♀ [on cards], China, Heilongjiang Province, Yichun City, Dailing District, Liangshui National Nature Reserve, 1.VIII.2015, Si-Zhu Li, Xin-Yu Zhang and Xing-Yue Jin, by sweeping.

**Figure 9.**
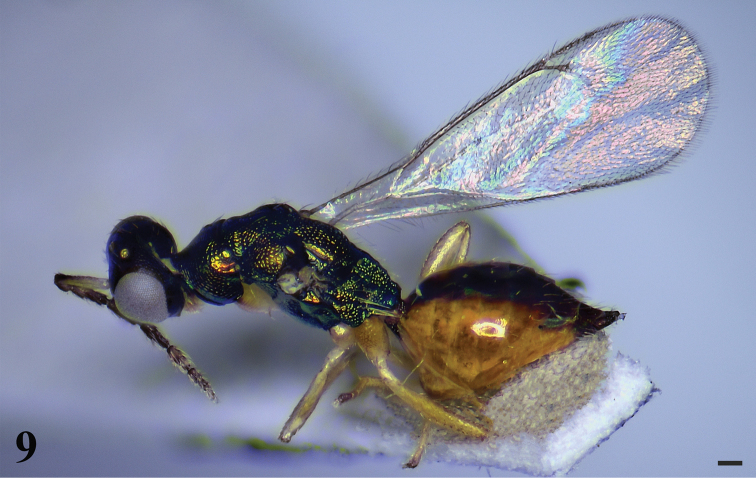
*Hemiptarsenuslongjiangensis* sp. nov., female, holotype, habitus in lateral view. Scale bar: 100 μm.

##### Diagnosis.

Scape yellow with about apical 1/3 of dorsal surface dark brown, pedicel and flagellum dark brown. Metascutellum reticulate. Propodeum almost as long as length of mesoscutellum measured medially, strongly reticulate, median carina and plicae present. Gaster with a large, median, longitudinal, black patch from base to apex, margins of tergites 1–5 yellow. Metasoma almost as long as mesosoma.

##### Description.

**Female.** Length 1.9–2.1 mm (2.1 mm), fore wing length 1.8 mm. Head and mesosoma dark metallic green with greenish-blue to golden-green tinge. Eyes gray. Ocelli pale yellow. Antennae dark brown except scape yellow with about apical 1/3 of dorsal surface dark brown. Mandibles brownish with teeth brown. Petiole dark brown. Gaster with a large median longitudinal black patch in middle of dorsal surface from base to apex, margins of tergites 1–5 yellow. Legs yellowish with all trochanters yellowish white. Ovipositor black. Wings hyaline with veins yellowish brown.

***Head*** (Fig. 11) 1.3–1.5× (1.5×) as wide as high in frontal view and about 1.8–2.0× (1.9×) as wide as long in dorsal view. Lower face and vertex transversely reticulate, frons weakly reticulate. POL 1.7–1.8× (1.8×) OOL. Eyes with sparse, short pubescence. Malar sulcus present, malar space 0.26 × eye height. Each mandible with two large teeth at apex and three small teeth above large teeth. Distance between toruli 0.8× diameter of torulus, 1.0× distance from torulus to eye margin. Antennae (Fig. 12) with scape slender and cylindrical, 5.6–5.7× (5.7×) as long as wide, extending far beyond vertex; pedicel 1.5–1.6× (1.6×) as long as wide and scape 3.6–3.7× (3.6×) as long as pedicel; funicle 4-segmented, F1 3.3–3.5× (3.5×) as long as wide and 2.2× as long as pedicel, F2 2.9–3.0× (3.0×) as long as wide, F3 and F4 2.4–2.6× (2.4×) and 1.8–2.1× (1.8×) as long as wide respectively; clava 2-segmented, 2.5–2.6× (2.5×) as long as wide, first clavomere 1.6–1.7× (1.6×) as long as second.

**Figures 10–15.**
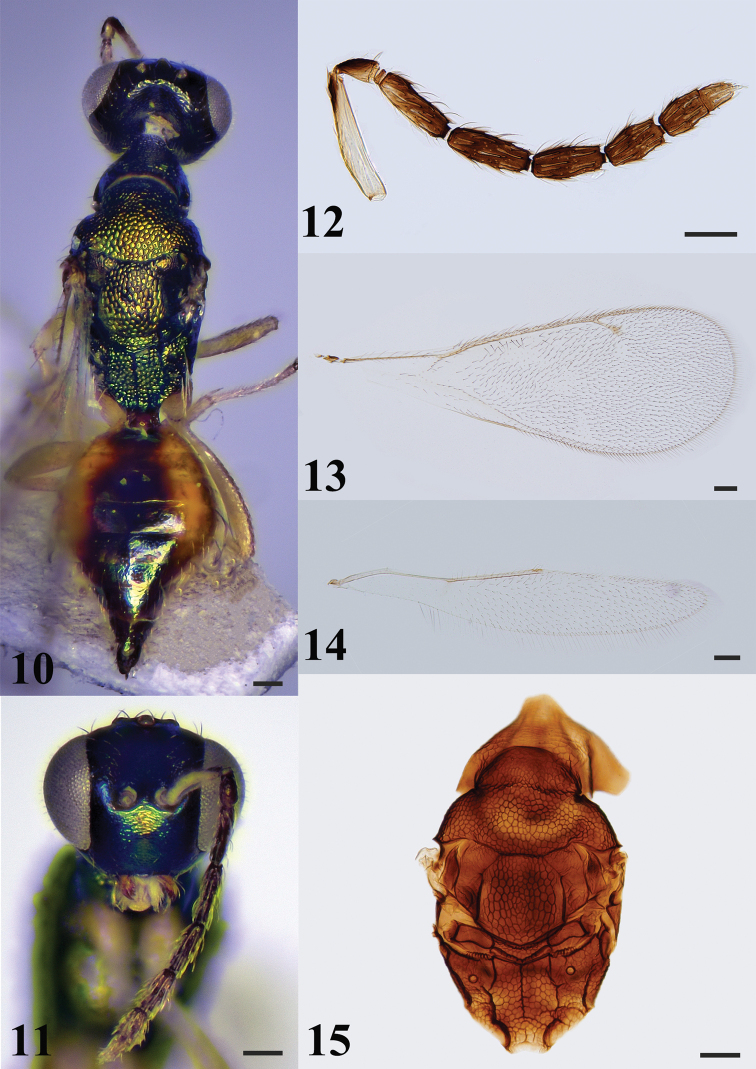
*Hemiptarsenuslongjiangensis* sp. nov., female, holotype (**10–14**), paratype (**15**) **10** habitus in dorsal view **11** head in frontal view **12** antenna **13** fore wing **14** hind wing **15** mesosoma in dorsal view. Scale bars: 100 μm.

***Mesosoma*** (Figs 10, 15) 1.8–2.0× (2.0×) as long as wide. Pronotum shorter than mesoscutum, reticulate. Notauli inconspicuous. Mesoscutum strongly reticulate, midlobe of mesoscutum with three pairs of long setae. Axillae reticulate and separate from each other. Mesoscutellum almost as long as wide, shorter than mesoscutum, strongly reticulate with two pairs of long setae. Metascutellum reticulate. Propodeum almost as long as length of mesoscutellum measured medially, strongly reticulate, median carina and plicae present; spiracle separated from metanotum by a distance longer than diameter of spiracle; each propodeal callus with nine setae. Prepectus with coarse reticulate sculpture. Metacoxa reticulate on dorsal surface.

***Wings*.** Fore wing (Fig. 13) 2.7–2.9× (2.8×) as long as wide. Costal cell 15.4–16.0× (16.0×) as long as wide. SMV with 10 setae on dorsal surface. Cubital vein straight at base. Speculum small, closed posteriorly. MV 1.3–1.4× (1.3×) as long as PMV; PMV 1.9–2.0× (1.9×) as long as STV. Hind wing (Fig. 14) about 5.7–2.9× (5.9×) as long as wide.

***Metasoma*** (Fig. 10) almost as long as mesosoma. Petiole short, transverse, about 0.5× as long as wide in dorsal view. Gaster ovate, 1.5–1.6× (1.5×) as long as wide. Ovipositor exserted beyond apex of gaster.

**Male.** Unknown.

##### Host.

Unknown.

##### Distribution.

China (Heilongjiang).

##### Etymology.

The specific epithet refers to Heilongjiang Province where the type locality is located.

#### ﻿ Hemiptarsenus jilinus

Taxon classificationAnimaliaHymenopteraEulophidae

Tao, 2021

757062E9-65A7-5E1C-854D-4A95B3FACB87

Figs 16–20

Hemiptarsenus jilinus Tao, 2021: 175. Holotype, ♀, China, IMJAU, not examined.

##### Material examined.

11♀ [NEFU; 10 on cards, 1 on slide], China, Liaoning Province, Fushun City, Shimengou, 18–20.VI.2012, Hui Geng, Xiang-Xiang Jin and Jiang Liu, by yellow-pan trapping; 1♀ [on card], China, Heilongjiang Province, Yichun City, Dailing District, Liangshui National Nature Reserve, 30. VIII. 2019, Wen-Jian Li, Ting-Ting Zhao and Shu-Chen Deng, by sweeping; 1♀ [on card], China, Heilongjiang Province, Yichun City, Dailing District, Liangshui National Nature Reserve, 30.VI.2018, Jun-Jie Fan, Guang-Xing Wang and Jun Wu, by sweeping; 2♀ [NEFU; 2 on cards], China, Beijing, Baihuashan, 10–11.V.2012, Hui-Lin Han, Guo-Hao Zu and Jiang Liu, by yellow-pan trapping; 2♀ [NEFU; 2 on cards], China, Hebei Province, Chengde City, Xinglong County, Wulingshan, 16–18.V.2017, Guang-Xing Wang and Wen-Jian Li, by yellow-pan trapping.

**Figures 16–20.**
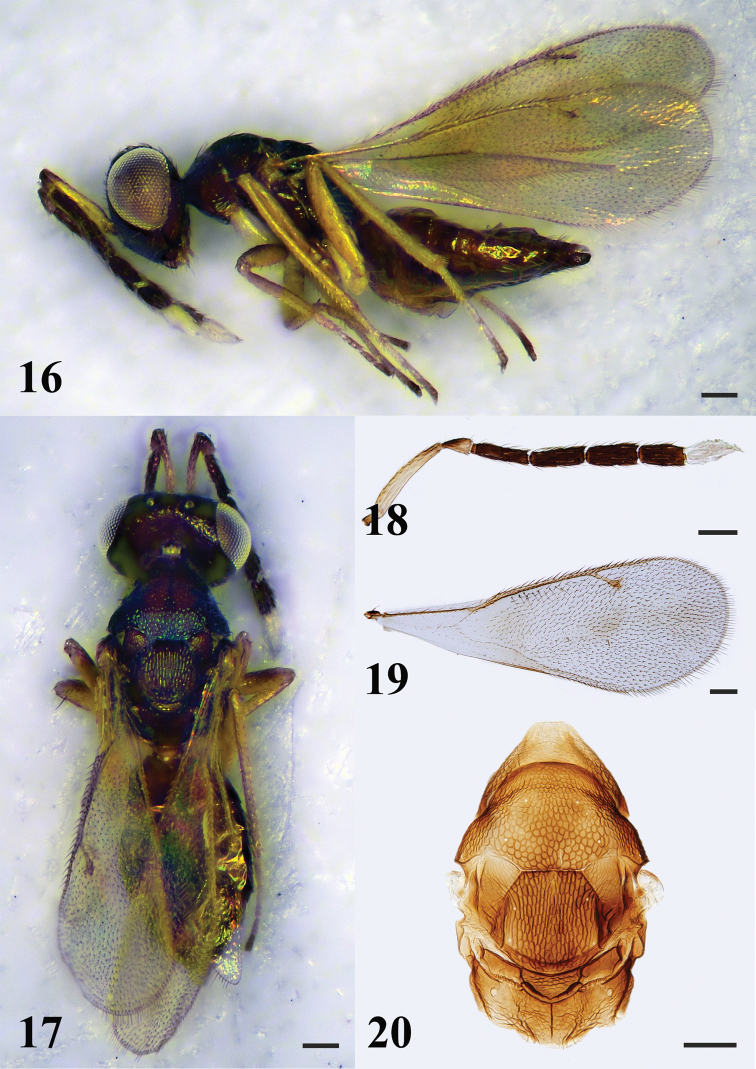
*Hemiptarsenusjilinus* Tao, female **16** habitus in lateral view **17** habitus in dorsal view **18** antenna **19** fore wing **20** mesosoma in dorsal view. Scale bars: 100 μm.

##### Diagnosis.

**Female.** Head and mesosoma dark metallic green; gaster brown with or without yellowish patch near base. Antennae with funicle dark brown, scape and pedicel pale yellow or with dark on dorsal surface, clava white. Legs yellow with coxae and trochanters white. Mesoscutellum longitudinally sculptured. Metascutellum raised-reticulate. Propodeum shorter than mesoscutellum, with median carina and plicae complete. POL 1.6–1.7× OOL. Malar sulcus present, malar space 0.4–0.5× eye height. Antennae (Fig. 18) with scape slender and cylindrical, 6.7–8.2× as long as wide, extending far beyond vertex; pedicel 1.6–1.8× as long as wide; funicle 4-segmented, F1 2.9–3.7× as long as wide, F2 3.4–4.1× as long as wide, F3 and F4 2.3–2.5× and 2.2–2.3× as long as wide respectively; clava 2-segmented, 2.4–2.6× as long as wide. Fore wing (Fig. 19) 2.6–2.8× as long as wide. Costal cell 13.3–13.7× as long as wide. Speculum present, closed posteriorly. MV 1.1–1.3× as long as PMV; PMV 1.6–1.8× as long as STV. **Male.** See Tao et al. (2021).

##### Host.

Primary parasitoid of *Chromatomyiahorticola* (Goureau) (Diptera, Agromyzidae) (Tao et al. 2021).

##### Distribution.

China (Jilin) (Tao et al. 2021); new records: Beijing, Heilongjiang, Liaoning, Hebei).

## Supplementary Material

XML Treatment for Hemiptarsenus tianshuiensis

XML Treatment for Hemiptarsenus longjiangensis

XML Treatment for Hemiptarsenus jilinus
